# Transcriptome analysis of *Valsa mali* reveals its response mechanism to the biocontrol actinomycete *Saccharothrix yanglingensis* Hhs.015

**DOI:** 10.1186/s12866-018-1225-5

**Published:** 2018-08-22

**Authors:** Cong Liu, Dongying Fan, Yanfang Li, Yue Chen, Lili Huang, Xia Yan

**Affiliations:** 1 State Key Laboratory of Crop Stress Biology for Arid Areas, Northwest A&F University, Yangling, Shaanxi China; 2 College of Life Science, Northwest A&F University, Yangling, Shaanxi China; 3 College of Plant Protection, Northwest A&F University, Yangling, Shaanxi China

**Keywords:** *Valsa Mali*, *Saccharothrix yanglingensis* Hhs.015, Response mechanism, Transcriptome analysis

## Abstract

**Background:**

Apple canker is a devastating branch disease caused by *Valsa mali* (*Vm*). The endophytic actinomycete *Saccharothrix yanglingensis* Hhs.015 (*Sy* Hhs.015) can effectively inhibit the growth of *Vm*. To reveal the mechanism, by which *Vm* respond to *Sy* Hhs.015, the transcriptome of *Vm* was analyzed using RNA-seq technology.

**Results:**

Compared with normal growing *Vm* in the control group, 1476 genes were significantly differentially expressed in the *Sy* Hhs.015’s treatment group, of which 851 genes were up-regulated and 625 genes were down-regulated. Combined gene function and pathway analysis of differentially expressed genes (DEGs) revealed that *Sy* Hhs.015 affected the carbohydrate metabolic pathway, which is utilized by *Vm* for energy production. Approximately 82% of the glycoside hydrolase genes were down-regulated, including three pectinase genes (PGs), which are key pathogenic factors. The cell wall structure of *Vm* was disrupted by *Sy* Hhs.015 and cell wall-related genes were found to be down-regulated. Of the peroxisome associated genes, those encoding catalase (CAT) and superoxide dismutase (SOD) which scavenge reactive oxygen species (ROS), as well as those encoding AMACR and ACAA1 which are related to the β-oxidation of fatty acids, were down-regulated. MS and ICL, key genes in glyoxylate cycle, were also down-regulated. In response to the stress of *Sy* Hhs.015 exposure, *Vm* increased amino acid metabolism to synthesize the required nitrogenous compounds, while alpha-keto acids, which involved in the TCA cycle, could be used to produce energy by deamination or transamination. Retinol dehydrogenase, associated with cell wall dextran synthesis, and sterol 24-C-methyltransferase, related to cell membrane ergosterol synthesis, were up-regulated. The genes encoding glutathione S-transferase, (GST), which has antioxidant activity and ABC transporters which have an efflux function, were also up-regulated.

**Conclusion:**

These results show that the response of *Vm* to *Sy* Hhs.015 exposure is a complicated and highly regulated process, and provide a theoretical basis for both clarifying the biocontrol mechanism of *Sy* Hhs.015 and the response of *Vm* to stress.

**Electronic supplementary material:**

The online version of this article (10.1186/s12866-018-1225-5) contains supplementary material, which is available to authorized users.

## Background

Apple canker is a serious and potential devastating branch disease caused by the ascomycetous fungus, *Valsa mali* (*Vm*), which occurs in the main apple producing areas of China [1], and causes serious economic losses. Currently, chemical treatment methods, such as scraping the canker lesion and applying fungicides, are the main strategies for preventing and treating apple canker [2]. However, large quantities of chemicals pollute the environment and can easily lead to drug-resistant pathogens. The usage of biological control agents has drawn increasing attention because they are environmental friendly, long-term and continuous [3].

*Vm* is a weak parasitic fungus that usually infects wounded or necrotic branches rather than healthy ones. *Vm* also has latent infection characteristics, as observed from the fact that decomposition of apple branches that look apparently free from disease can occur after specific treatments [4]. The process of *Vm* infecting apple trees is complicated, and cell wall- degrading enzymes, secondary metabolites, and effector proteins might play important roles in their pathogenic mechanism [5]. During the process of infecting apple bark, the expression of genes related to catabolism, hydrolase activity and secondary metabolite biosynthesis are up-regulated [6]. Additionally, the use of immunocytochemistry labeling has shown that pectinases play an important role in the infection process [6]. *Vm* can also produce toxins such as protocatechuic acid, p-hydroxybenzoic acid, p-hydroxyacetophenone, 3-p-hydroxyphenylpropionic acid and phloroglucinol [7]. Some genes related to toxin synthesis have been identified in the genome of *Vm*, and genes related to secondary metabolism such as cytochrome P450, non-ribosomal polypeptide synthetase and monooxygenase, have been shown to be up-regulated during infection [5].

Actinomycetes are a class of microbes that are kown to produce bioactive substances [8]. They are of potential value to biocontrol because they can inhibit pathogens by producing natural products such as antibiotics and extracellular enzymes [9]. The *Saccharothrix yanglingensis* strain Hhs.015 (*Sy* Hhs.015) is an endophytic actinomycete isolated from the root of cucumber [10]. Both laboratory and field experiments have proven that *Sy* Hhs.015 is a good inhibitor of apple canker. In vitro experiments have shown that *Sy* Hhs.015 sterile fermentation filtrate can inhibit the growth of mycelium and conidia germination of *Vm*, and abnormal mycelia and cytoplasmic extravasation can be observed. Field experiments has shown that the relative control efficiency of apple trees infected with *Vm* after *Sy* Hhs.015 treatment was 61.29%, which was equivalent to that of treatment with difenoconazole and tebuconazole [11]. Studies have shown that *Sy* Hhs.015 can produce heteroauxin, chitinase, proteinase and glucanase, and the active substances isoflavones and pentamycin have been extracted from its fermentation broth [11].

This study aims to elucidate determine the changes in cell structure and gene expression level in *Vm* upon challenged with *Sy* Hhs.015. Therefore, RNA-seq was used to compare the gene expression of normal *Vm* with treated with *Sy* Hhs.015.

## Results

### Inhibition of *Vm* by *Sy* Hhs.015

**Fig. 1:**
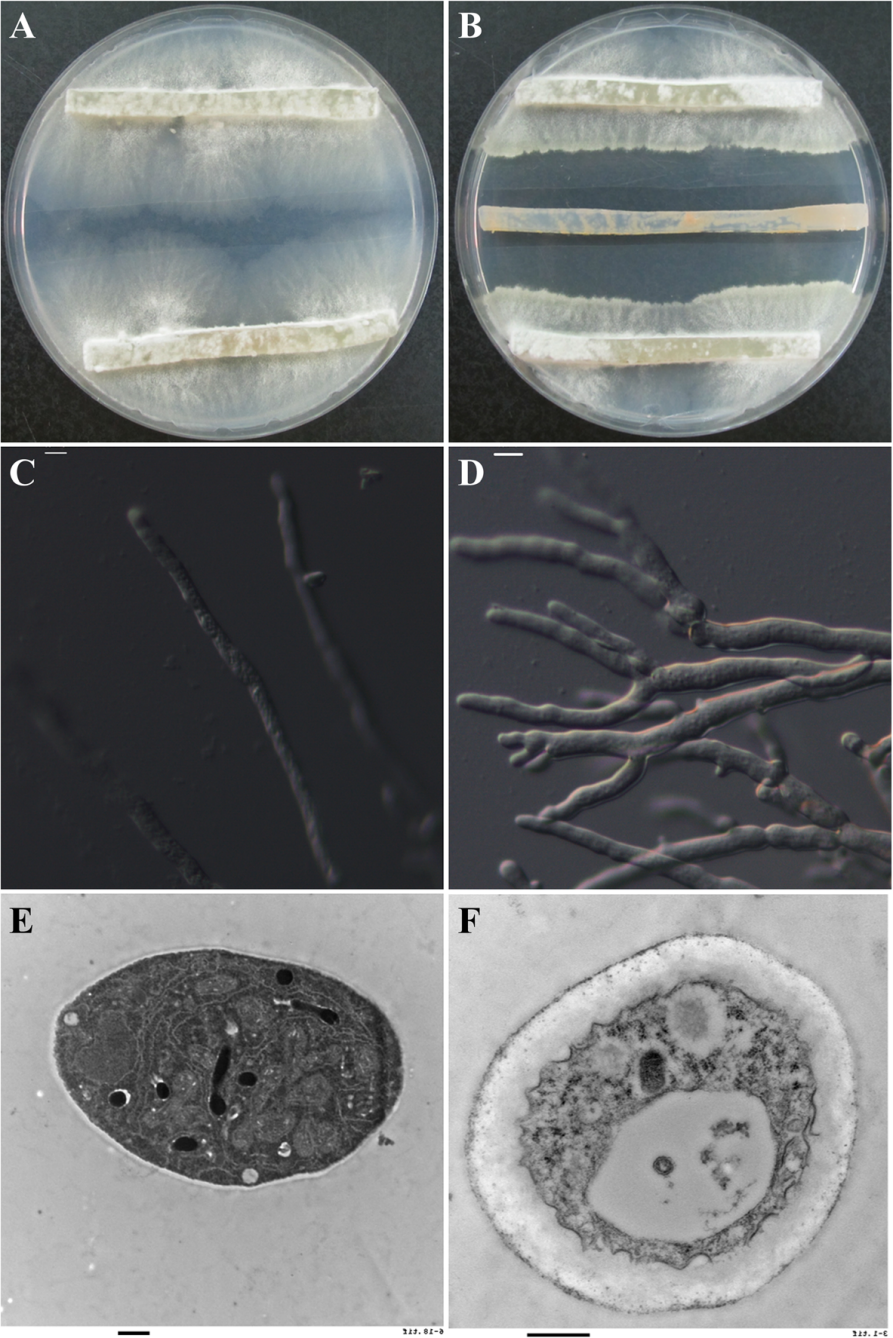
Graphs of *Vm* treated with *Sy* Hhs.015 for 48 h. **a** Normal growth of *Vm.* **b** *Vm* treated with *Sy* Hhs.015. Mycelial morphology observed by optical microscope, bar = 10 μm: **c** Normal hyphae. **d** Abnormal hyphae with branches. Subcellular structure of mycelium observed by transmission electron microscope, bar = 500 nm: **e** Normal *Vm* showed a clear and complete cell structure. **f** Treated *Vm* showed thickening of cell walls and organelle degradation

The *V. mali*/*S. yanglingensis* confrontation assay showed that at 48 h *Sy* Hhs.015 significantly inhibited the growth of *Vm* mycelia (Fig. 1a and b). Additionally, quantities of abnormal mycelia could be observed using an optical microscope (Fig. 1c and d). Transmission electron microscopy of the subcellular structure of abnormal mycelia showed that the cell wall had thickened, almost all the cytoplasm had degenerated completely and a large vacuole had formed. In addition, the nucleus had fully separated from the cytoplasm, while several cells had even completely degraded in the nucleus (Fig. 1e and f).

### Sequencing quality control, quantification of gene expression levels, and annotation of gene function

The quality of extracted RNA reaches the standard of sequencing, and the mass concentration of each sample is higher than 36 ng/μL (Additional file 1: Table S1). The total number of raw reads obtained by sequencing was 133,185,208, while the total number of clean reads after filtration was 126,932,206. The total number of clean reads bases was 15.86 Gb. The error rate of each sample was less than 0.04%, Q20 was greater than 94.24%, Q30 was greater than 89.06%, and GC content was 55.02–55.84%. The raw data of all samples (three controls and three treatments) reported in this study have been deposited in the Genome Sequence Archive [12] in BIG Data Center [13], Beijing Institute of Genomics (BIG), Chinese Academy of Sciences, under accession number CRA000693, which is publicly accessible at http://bigd.big.ac.cn/gsa. Sequencing data showed that quality and accuracy were both excellent (Table 1). The mapping rate of each sample was higher than 96.4%. The count matrix for each sample was also obtained (Additional file 2: Table S2).

**Table 1:** Statistics of sequencing production and mapping ratio

Sample	Raw reads	Clean reads	Clean bases	Q30(%)	GC(%)	Total mapped Ratio (%)
C1_1	11,645,164	10,899,898	1.36G	92.45	55.84	96.89
C1_2	11,645,164	10,899,898	1.36G	89.78	55.82	
C2_1	10,609,958	10,166,658	1.27G	92.22	55.05	96.97
C2_2	10,609,958	10,166,658	1.27G	89.50	55.03	
C3_1	10,455,527	9,917,147	1.24G	92.70	55.04	96.79
C3_2	10,455,527	9,917,147	1.24G	89.06	55.02	
T1_1	10,848,694	10,403,574	1.30G	93.49	55.35	96.56
T1_2	10,848,694	10,403,574	1.30G	91.03	55.37	
T2_1	11,187,720	10,767,189	1.35G	93.54	55.12	96.65
T2_2	11,187,720	10,767,189	1.35G	90.88	55.13	
T3_1	11,845,541	11,311,637	1.41G	92.72	55.19	96.47
T3_2	11,845,541	11,311,637	1.41G	89.50	55.16	

KOG (Eukaryotic Ortholog Groups) [14] analysis divided homologous genes from different species into different ortholog clusters according to their evolutionary relationship. There were 25 groups of KOG annotations for 8359 *Vm* genes (Additional file 3: Figure S1), of which 2568 genes were annotated as “[S] Function unknown.” The top three groups by number of genes were “[G] Carbohydrate transport and metabolism (8.52%),” “[O] Posttranslational modification protein turnover chaperones (6.56%),” and “[Q] Secondary metabolites biosynthesis transport and catabolism (6.29%).” However, the three groups with a small number of genes are the “[W] Extracellular structures (0.05%),” “[N] Cell motility (0.04%),” and “[Y] Nuclear structure (0.04%)”.

GO (Gene Ontology) was defined according to the molecular functions, biological pathways, and cytological components of the gene product [15]. Among 11,284 gene sequences in the *Vm* genome, 7332 genes had GO annotations, which were classified into 32 categories (Additional file 4: Figure S2). In the class “biological process,” the two most populated categories were “metabolic process” and “cellular process.” In the class “cellular component,” the three most populated categories were “cell,” “cell part,” and “organelle.” In the class “molecular function,” the two most populated categories were “catalytic activity” and “binding.” In contrast, there was only one gene in each of the categories of “cell proliferation,” “locomotion,” “pigmentation,” and “extracellular region part,” respectively.

KEGG (Kyoto Encyclopedia of Genes and Genomes) is a database that systematically analyzes the metabolic pathways of gene products and compounds in cells as well as the function of these gene products [16]. There were 3635 genes with KEGG annotations divided into 24 categories (Additional file 5: Figure S3). In addition to the “Global and overview map” category, the seven most populated categories were “Carbohydrate metabolism,” “Amino acid metabolism,” “Translation,” “Signal transduction,” “Transport and catabolism,” and “Cell growth and death.” While the number of genes in the category of “Membrane transport” and “Signaling molecules and interaction” totaled only 0.16% and 0.04% of the genome respectively.

### Differential expression analysis and GO, KEGG enrichment analysis

**Fig. 2:**
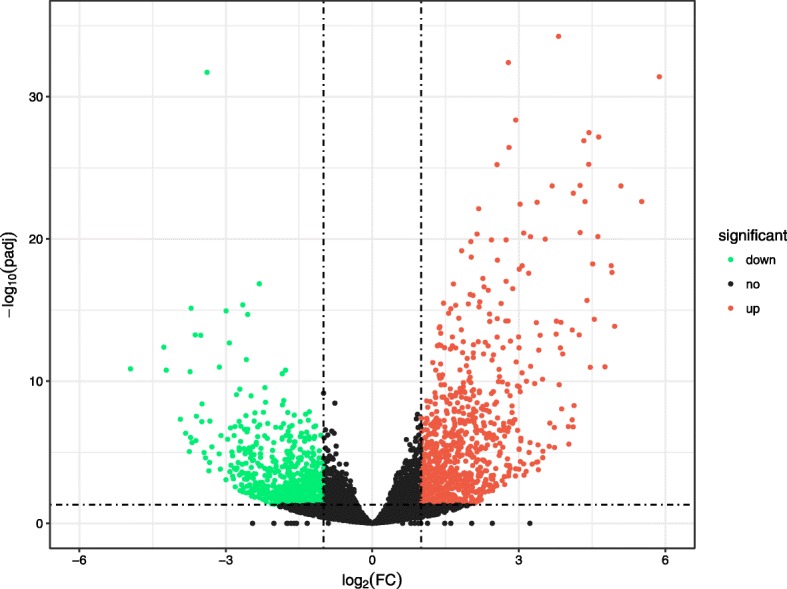
Volcano plot of DEGs between *Sy* Hhs.015 treatment group and control group. With log2 (Fold Change) as the x-axis and log10 (p.adj) as the y-axis, the volcano plot was made according to the gene expression level. The red dots indicate up-regulated genes, the green dots indicate down-regulated genes and the black dots indicate non-significant differentially expressed genes

Differentially expressed genes were calculated according to the gene expression count matrix using R package DESeq2. 1476 DEGs with |log2FC| ≥ 1 and *P*.adj < 0.05 were obtained from the *Sy* Hhs.015 treatment group compared with the control group, among which 851 genes were up-regulated and 625 genes were down-regulated. A volcano plot was constructed according to gene expression level (Fig. 2).

**Fig. 3:**
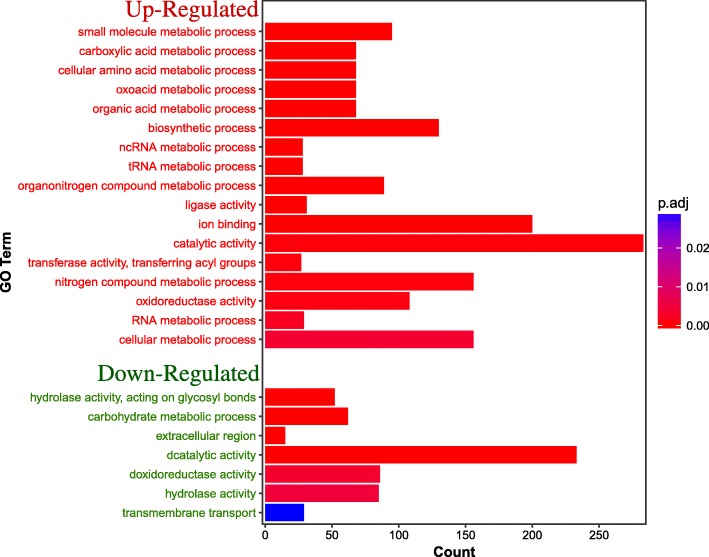
Bar plot of GO enrichment analysis of DEGs. “Count” indicates the number of DEGs enriched in GO term. And “p.adj” indicates the *p*-value corrected by “BH” method

Gene set enrichment analysis was performed to find groups of genes or proteins that are extract over-expressed. GO enrichment analysis was performed to reveal the relationship between the function of DEGs and the response to *Sy* Hhs.015 treatment. The results showed that there were 17 significantly enriched terms (*p*.adj < 0.05) for up-regulated genes (Fig. 3). Down-regulated genes were significantly enriched in 5 terms (*p*.adj < 0.05).

**Fig. 4:**
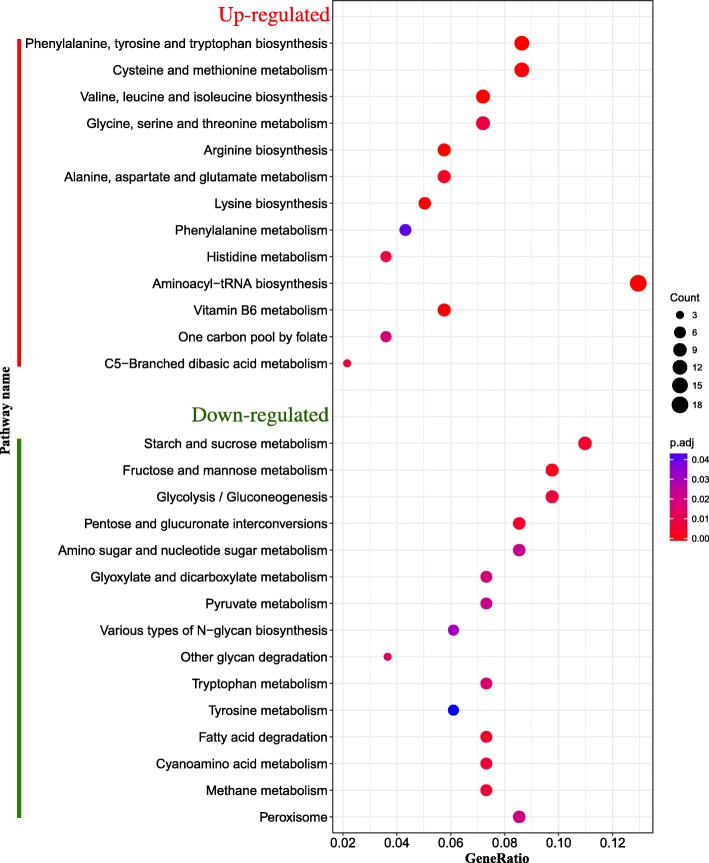
Bubble plot of KEGG pathway enrichment analysis of DEGs. “Count” indicates the number of DEGs enriched in pathway. “GeneRatio” indicates the ratio of enriched DEGs to background genes. And “p.adj” indicates the *p*-value corrected by ‘BH’ method

KEGG pathway enrichment analysis helps us to analyze gene and expression networks. Pathway enrichment analysis showed that up-regulated genes were significantly enriched in 13 pathways (*p*.adj < 0.05) (Fig. 4), of which 78 genes were enriched in the “Amino acid metabolism (ko00400, ko00290, ko00300, ko00220, ko00270, ko00250, ko00340, ko00260, ko00360)” pathway. In addition, 18 genes were enriched in the “Translation (ko00970)” pathway and 13 genes were enriched in the “Metabolism of cofactors and vitamins (ko00750, ko00670)” pathway. However, only 3 genes were involved in the pathway “Carbohydrate metabolism (ko00660).” Down-regulated genes were significantly enriched in 15 pathways (*p*.adj < 0.05), most of which were involved in the “Carbohydrate metabolism (ko00051, ko00040, ko00500, ko00010, ko00630, ko0520, ko00620)” pathway. Pathways related to “Glycan biosynthesis and metabolism (ko00511, ko00513),” “Fatty acid degradation (ko00071),” and “Peroxisome (ko04146)” were also significantly enriched.

### Carbohydrate-active enzymes of DEGs

**Fig. 5:**
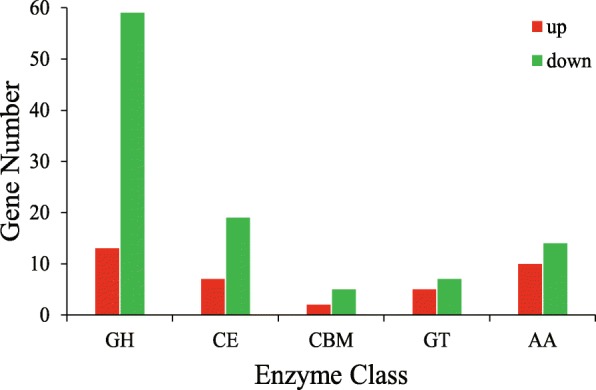
Statistics of up-regulated and down-regulated CAZymes in DEGs. The x-axis indicates the classification of CAZymes, and the y-axis indicates the number of genes

**Fig. 6:**
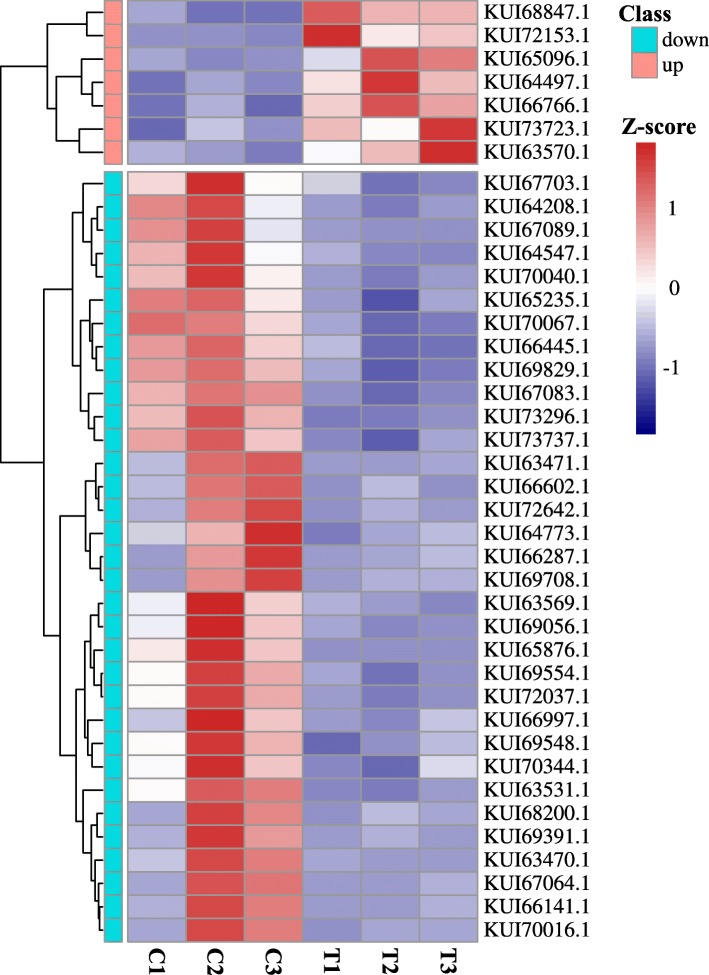
Heatmap of extracellular glycosyl hydrolases. The color scale indicates the counts of gene expression normalized by Z-score. “C1, C2, and C3” indicates the three replicates of the control group. “T1, T2, and T3” indicates the three replicates of the treatment group

Carbohydrate-active enzymes (CAZymes) are responsible for the synthesis and metabolism of carbohydrates. CAZymes are often involved in plant pathogens and host interactions [17]. By homology alignment of the CAZy database, 72 Glycoside Hydrolases (GHs), 26 Carbohydrate Esterases (CEs), 7 Carbohydrate-Binding Modules (CBMs), 12 Glycosyl Transferases (GTs) and 24 Auxiliary Activities (AAs) were found in 1476 DEGs (Fig. 5). It is noteworthy that 33 out of 40 (~ 82%) extracellular GH genes were down-regulated (Fig. 6).

### qRT-PCR validation

**Fig. 7:**
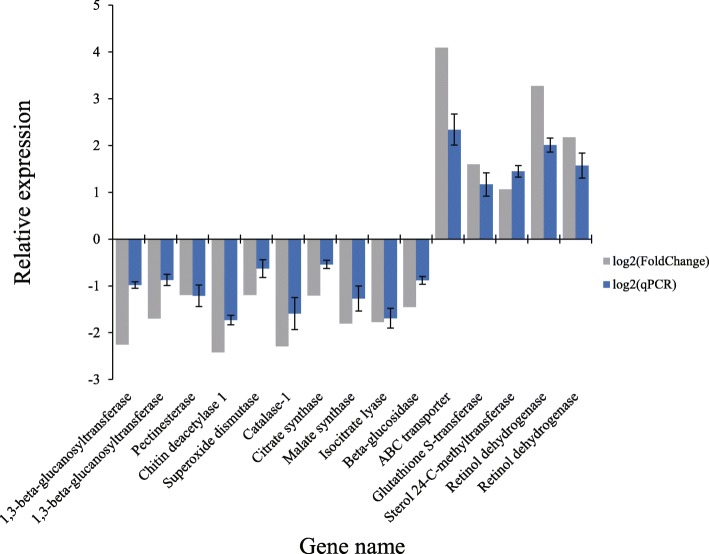
Relative expression level of fourteen DEGs using reference gene G6PDH for normalization. The x-axis indicates the name of the DEGs, and the y-axis indicates the relative expression of the DEGs

The functions of 15 DEGs for qPCR include carbohydrate metabolism, synthesis and repair of cell wall and membrane structure, antioxidant and detoxification, glyoxylate cycle and TCA cycle, and pathogenicity of *Vm*. The qPCR results showed that the actual expression of 15 DEGs was consistent with the trend of gene expression obtained by analysis, but there was a difference in the relative expression level of the genes, which may due to the difference between qPCR technique and the calculation method of differential expression analysis (Fig. 7).

## Discussion

In this study, we observed that *Sy* Hhs.015 significantly inhibited the growth of *Vm*. At the subcellular level, the mycelia of *Vm* were distorted and branched. Additionally, we observed extravasation of the protoplasm and the disruption of cellular structure. These results are consistent with previous reports [2]. The chitinase, glucanase and protease produced by *Sy* Hhs.015 can destroy the cell wall of *Vm*. The phenylacetic acid isolated from the fermentation broth of *Sy* Hhs.015 can also inhibit *Vm*. Comparison of the expression of genes from *Vm* cell that had been inhibited by *Sy* Hhs.015 with that of cells from *Vm* cells, revealed 1476 DEGs.

Our analysis of DEGs revealed that the carbohydrate metabolism of *Vm* had been greatly influenced by *Sy* Hhs.015. In the KOG annotation of DEGs, there were 95 down-regulated genes and 47 up-regulated genes in the group “[G] Carbohydrate transport and metabolism.” GO annotation showed that the category “Carbohydrate metabolic process” significantly enriched 62 down-regulated genes (*p*.adj = 4.55e-14), and the category “hydrolase activity” significantly enriched 85 down-regulated genes (*p*.adj = 0.0045). The same effect was evident in the large number of glycoside hydrolase genes that showed down-regulated expression. Pectinase genes (PGs) are key virulence factors for phytopathogenic fungi, which can impair the pectin network of plant cell walls and participate in the maceration of tissues during fungal infection [18]. Three PGs (KUI67703.1, KUI69548.1, KUI73936.1) among the DEGs were down-regulated, which may affect *Vm* infectivity. Carbohydrate metabolism is an important way for the organism to gain energy [19]. Heterotrophic fungi, for example, usually gain nutrients by secreting extracellular hydrolase. The hydrolases secreted by pathogenic fungi can damage plant cell wall by breaking down polysaccharides, thereby facilitating infection [20]. Decreased carbohydrate metabolism reduces the production of acetyl-CoA. And the key enzyme citrate synthase in the TCA cycle is down-regulated. All of these factors lead to *Vm* gained energy shortage.

Chitin, dextran and various proteins are important components of the fungal cell wall [21]. Interestingly, four of the five genes associated with cell walls in DEGs were down-regulated, while eight down-regulated genes appeared in the “Glycan biosynthesis and metabolism” pathway. One Chitin deacetylase 1 gene (KUI65489.1) that played a role in cell wall chitosan biosynthesis was also down-regulated (log2FC = − 2.42) [22]. It could be inferred that the ability to biosynthesize the polysaccharide components in the cell wall had decreased. Chitinase is an important enzyme that degrades cell compartments and achieves cell separation during fungal proliferation [23]. Two Chitinase 1 genes (KUI66287.1, KUI69708.1) among the DEGs were significantly down-regulated (log2FC = − 2.21). Meanwhile, chitinase, proteinase and glucanase produced by *Sy* Hhs.015 also destroyed the cell wall structure of *Vm* [11]. Combining these factors, it can be speculated that *Sy* Hhs.015 damaged the cell wall of *Vm* and cell wall formation and cell division were blocked, resulting in the inhibition of *Vm* growth.

Peroxisomes are a type of monolayer organelle commonly found in eukaryotes, and contain oxidase, catalase, and peroxidase [24]. Catalase is a peroxidase marker enzyme, and its main function is to hydrolyze the cytotoxic substance H_2_O_2_ produced in oxidase catalyzed redox reactions. Seven down-regulated genes were significantly enriched in the “Peroxidase” pathway (*p*.adj = 0.0196), including a catalase gene (KUI65198.1, log2FC = − 2.29) and a superoxide dismutase gene (KUI66682.1, log2FC = − 1.19). These changes may not only affect the oxidation of toxic substances such as formic acid and phenol, but also accumulate H_2_O_2_, leading to cell damage. The pathogen’s fungal glyoxylate cycle are involved in its infection process [25]. Malic acid synthase (MS) and Isocitrate lyase (ICL) are the key enzymes in the glyoxylate cycle, both of which are also present in the peroxisome. Down regulation of the two genes may also reduce the use of acetyl-CoA through the glyoxylate cycle. Medium-chain fatty acids produced by the beta-oxidation of fatty acids affect the production of pigments and toxins in fungi. In addition, acetyl coenzyme A, another product, is both essential for the infection process and promotes gluconeogenesis [26]. Two down-regulated genes (KUI71646.1, “alpha-methylacyl-CoA racemase,” log2FC = − 1.34; KUI64199.1, “acetyl-CoA acyltransferase 1,” log2FC = − 1.15) of β-oxidation related to fatty acid may affect the fatty acid metabolism and the pathogenicity of *Vm*.

Organisms can respond to external stress through their own regulation mechanisms [27]. The pathways “Amino acid metabolism (78 up-regulated genes)” and “Aminoacyl-tRNA biosynthesis (18 up-regulated genes),” that are related to translation and involved in amino acid biosynthesis, were significantly enriched [28]. The pathway “Vitamin B6 metabolism (6 up-regulated genes)” was also enriched. Vitamin B6 can participate in amino acid, glucose, and lipid metabolism via its metabolically active form, pyridoxal 5′-phosphate (PLP) [29]. Furthermore, the KOG annotation also showed that amino acid biosynthesis and metabolism had been enhanced, including the groups “[O] Posttranslational modification protein turnover chaperones (36 up-regulated, 18 down-regulated)” and “[E] Amino acid transport and metabolism (68 up-regulated, 29 down-regulated).” The amino acid biosynthesis and metabolism pathways have two beneficial purposes: the synthesis of the proteins needed for survival and the generation of α-keto acids, through deamination and transamination, which can then participate in carbohydrate metabolism, lipid metabolism and the TCA cycle to obtain energy. Alanine, aspartate, glutamate and histidine metabolism can produce 2-oxoglutarate and oxaloacetate. Degradation of valine, leucine and isoleucine can produce succinyl-CoA. Tyrosine metabolism and arginine biosynthesis can produce fumarate. These products can supplement the TCA cycle affected by down regulation of citrate synthase. For instance, glutamate generates α-ketoglutaric acid, involved in TCA cycle, which is catalyzed by glutamate dehydrogenase [30], and can compensate for a lack of carbohydrate metabolism. Four retinol dehydrogenase genes were up-regulated: these are associated with dextran synthesis [31] and may be involved in the stress repair process after the cell wall destruction of *Vm*. Ergosterol is an important component of the cell membrane, and interestingly, we found that the sterol 24-C-methyltransferase gene involved in its synthesis was up-regulated [32]. Glutathione S-transferase (GST) functions in the processes of detoxification and anti-oxidation and can also catalyze the binding of GSH^−^ to electrophilic centers on toxic substrates through sulfhydryl groups. Likewise, ABC transporters have the efflux function of excreting toxic substances [33]. Two GST genes (KUI68053.1, KUI73914.1) and two ABC transporters genes (KUI66519.1, KUI68518.1) were up-regulated, which most likely contributed to the detoxification and anti-oxidation of *Vm*. We also found that the Streptothricin hydrolase gene (KUI73345.1, log2FC = 3.38) was significantly up-regulated, which might indicate a counter response of *Vm* to antimicrobial substances such as antibiotics produced by *Sy* Hhs.015.

**Fig. 8:**
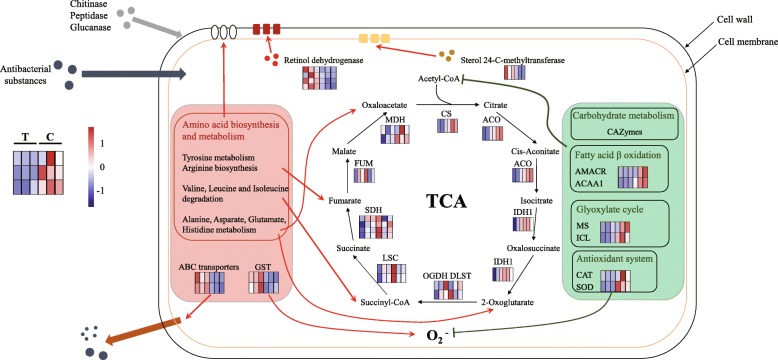
The speculative model diagram of *Vm* response to *Sy* Hhs.015. Heatmap shows gene expression. The color scale indicates the counts of gene expression normalized by Z-score

Combining the above analysis, we have speculated the response mechanism of *Vm* to *Sy* Hhs.015 (Fig. 8). A variety of antimicrobial substances produced by *Sy* Hhs.015 affected the carbohydrate and fatty acid metabolism pathways in *Vm* for energy, especially the utilization of acetyl-CoA. The cell wall and membrane structure of *Vm* was also destroyed. The catalase and superoxide dismutase genes responsible for the scavenging of oxygen free radicals in *Vm* cells are down-regulated, and accumulation of toxic substances can lead to cell damage. Not surprisingly, *Vm* has made multiple responses to inhibition of stress. *Vm* enhanced amino acids metabolism to compensate for the lack of energy acquisition through the production of alpha-keto acids, oxaloacetate, succinyl-CoA, and fumarate involved in TCA cycle. The up-regulated expression of retinol dehydrogenase genes involved in dextran biosynthesis as well as the sterol 24-C-methyltransferase gene, which are involved in the synthesis and repair of cell walls and cell membranes. Additionally, the GST and ABC transporter genes were up-regulated to increase antioxidant function and the ability of *Vm* to excrete extracellular substances.

In order to verify these speculations, qPCR tests for 15 DEGs associated with the model have been completed. In the future study, we will gradually complete the qPCR verification of the genes related to the prediction model. And it is also extremely necessary to explore the impact of complex plant environments on the results.

## Conclusion

In conclusion, we have initially shed light on the response mechanism of *Vm* to *Sy* Hhs.015. Many gene expression levels have changed in samples treated with *Sy* Hhs.015. *Sy* Hhs.015 can destroy the cellular structure of *Vm* and impair the TCA cycle and glyoxylate cycle. In order to respond to the inhibition of *Sy* Hhs.015, *Vm* enhances synthesis and metabolism of amino acids, synthesis and repair of cell walls and cell membranes, and detoxification and antioxidation. Overall, the results of this research provide a theoretical basis for clarifying the biological control mechanism of *Sy* Hhs.015 and the response mechanism of *Vm* to stress.

## Methods

### Strains and culture conditions

*Saccharothrix yanglingensis* strain Hhs.015 and *Valsa mali* virulent strain 03–8, were provided by the Laboratory of Integrated Management of Plant Diseases, College of Plant Protection, Northwest A&F University, Yangling, Shaanxi Province, China and sorted at − 80 °C.

*Vm* 03–8, on potato dextrose agar (PDA) and incubated at 25 °C for 3 days. *Sy* Hhs.015 was cultured on Gause’s No.1 synthetic agar medium and incubated at 28 °C for 7 days in the dark to induce sufficient sporulation.

### *V. mali*/*S. yanglingensis* confrontation assay

**Fig. 9:**
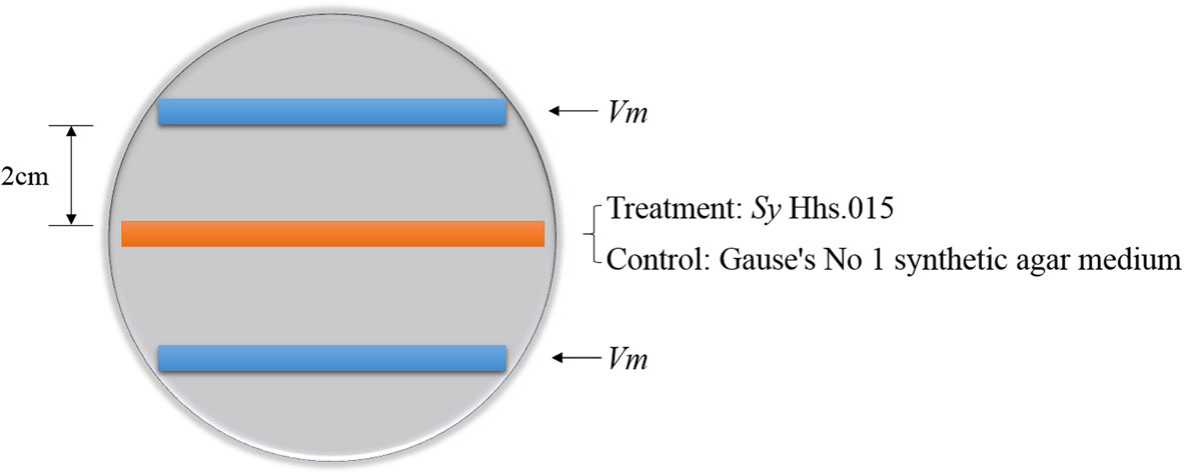
Schematic diagram of *Vm* /*Sy* Hhs.015 confrontation assay

A section of agar 0.5 cm in width and 8.5 cm in length was taken from a plate of Gause’s No.1 agar previously inoculated with *Sy* Hhs.015 and placed on the middle of a fresh PDA plate covered with sterile cellophane. Another section of agar 0.5 cm in width and 7.0 cm in length was taken from a plate of PDA inoculated with *Vm* and placed on the new PDA plate, 2 cm from *Sy* Hhs.015 agar strip (Fig. 9). After culture at 25 °C for 48 h in the dark, the mycelia at the *Sy* Hhs.015-exposed boundary of the *Vm* culture were collected and labeled as the treatment group. As a control, mycelia were collected from unexposed *Vm*. The experimental sample had three biological replicates. Samples were lyophilized with liquid nitrogen and stored at − 80 °C.

### RNA extraction and sequencing

Total RNA of the samples was extracted using the RNeasy Micro kit (Qiagen, Shenzhen, PRC). RNA degradation and contamination of the samples were assessed on 1% agarose gels. RNA purity was analyzed using a NanoPhotometer® spectrophotometer (IMPLEN, CA, USA). RNA concentration was measured using the Qubit® RNA Assay Kit in a Qubit® 2.0 Fluorometer (Life Technologies, CA, USA). RNA integrity was assessed using the RNA Nano 6000 Assay Kit of the Agilent Bioanalyzer 2100 system (Agilent Technologies, CA, USA).

A total of 3 μg of RNA per sample was used as input material for the RNA sample preparations. Sequencing libraries were generated using NEBNext® Ultra™ RNA Library Prep Kit for Illumina® (NEB, USA) following manufacturer’s recommendations and index codes were added to attribute sequences to each sample. The library preparations were sequenced on an Illumina Hiseq 2000 platform, where 125 bp paired-end reads were generated.

### Raw read cleaning, mapping to reference genome, and gene annotation

Trimmomatic (Version 0.36) was applied to obtain high-quality clean reads by trimming and filtering raw reads [34], removing adaptor sequences, low quality sequences and short reads (< 25 bp). The clean data for each sample was mapped to the *Vm* reference genome (NCBI ACCESSION: JUIY00000000) using default parameters of HISAT2 (Version 2.1.0) [35]. Using HTseq-count (Version 0.8.0), the number of reads mapped to each gene was calculated based on the SAM / BAM alignment result file and the GTF file of the gene structure to obtain a count matrix for differential expression analysis [36].

Blastp (2.4.0, E-value <1e-5) was used to align all genes with Nr, KOG, KEGG databases for functional annotation. The GO (Gene Ontology) annotation of the genes were obtained using Blast2GO (version 4.1) software [37].

### Differential expression analysis, GO, and KEGG enrichment analysis

Differentially expressed genes were identified between the *Vm* samples that were inhibited by *Sy* Hhs.015, and untreated *Vm* samples using R-package DESeq2 (Version 1.10.1) [38]. The package DESeq2 provides methods to test differential expression by using negative binomial generalized linear models. Log2-fold change, *p*-value and adjusted p-value were calculated for all genes. Genes with a |log2FC| ≥ 1 and *P*.adj < 0.05 were considered to be differentially expressed genes (DEGs).

Using GO and KEGG annotations of all genes in the *Vm* genome as background, GO and KEGG pathway enrichment analysis of DEGs was performed using a hypergeometric distribution test [39].

### Quantitative reverse transcription-PCR (qRT-PCR)

To confirm the reliability of DEGs, 15 genes were selected for qRT-PCR validation, while the endogenous gene glucose-6-phosphate-dehydrogenase (G6PDH) was used as a control. Primer Premier (Version 5.0) was used to design primers for the 15 selected DEGs (Table 2). PCR amplification was performed using the BIO-RAD system and the expression analysis was carried out using built-in software. The reaction system consisted of 1 μL of cDNA, 0.5 μL of 10 μM PCR primer, SYBR Premix ExTaq (1×, 10 μL; TaKaRa Bio Inc.) as a total of 20 μL. PCR program was as follows: 95 °C for 1 min, 40 cycles (95 °C for 15 s, 55 °C for 20 s, and 72 °C for 45 s). A dissolution curve was then generated. The qPCR for each gene was repeated 3 times, and the average (Ct) was calculated. The relative expression level of each gene was calculated using the 2^-ΔΔCt^ method [40].

**Table 2:** Primers used in qRT-PCR

Gene id	Function			Primer	
KUI72642.1	1,3-beta-glucanosyltransferase	F^a^	5’	CCGAGAAATACATGACCAAGGG	3’
		R^b^	5’	CAGTGCTAGTTCCAGATCCAG	3’
KUI73737.1	1,3-beta-glucanosyltransferase	F	5’	TGACTTCGCCAACCTCAAG	3’
		R	5’	TCACCATCGACTAACCATGC	3’
KUI73936.1	Pectinesterase	F	5’	ATCGAGGGTGTTACGGATTTC	3’
		R	5’	AGAGGACTTGCGACCATTG	3’
KUI65489.1	Chitin deacetylase 1	F	5’	AGAAAGCCCTTATCCGCAAG	3’
		R	5’	GCCCATCTCTTTATAGACCTCG	3’
KUI66682.1	Superoxide dismutase	F	5’	AGGAAATGTGAAGGGTGCC	3’
		R	5’	GTGGATGTGGTAGAGGAAAGG	3’
KUI65198.1	Catalase-1	F	5’	ATGGCTGTTTCCCTAAGTGG	3’
		R	5’	GCTCCAATTCACCTACATACCG	3’
KUI64284.1	Citrate synthase	F	5’	TTATGGATTACGCCTCAGCTC	3’
		R	5’	GGGTAGGGATTCTTGGTCTTG	3’
KUI74469.1	Malate synthase	F	5’	GTGAGGGCTGATAAGTTGAGG	3’
		R	5’	GCTGATTTGGTGTTGGCATG	3’
KUI72900.1	Isocitrate lyase	F	5’	GTGAACCCCGAGACAGAAG	3’
		R	5’	TCTTGGACACAATCTGCTCG	3’
KUI64411.1	Beta-glucosidase	F	5’	TGGACCTTCACAGATAATTGGG	3’
		R	5’	GAAGTCTACCCAAGTTACGCC	3’
KUI66519.1	ABC transporter	F	5’	TCACCTATGCAAAGAGATGGG	3’
		R	5’	GCCTGCCGAAAATGACATTC	3’
KUI68053.1	Glutathione S-transferase	F	5’	TGTGTTGCGGTATCTGAAGG	3’
		R	5’	AGCTTCGAACTGGGTGAAAC	3’
KUI70005.1	Sterol 24-C-methyltransferase	F	5’	ACATGCTGATCAACTTCCCTG	3’
		R	5’	GTACTTCTCAATACCCCATCCG	3’
KUI71751.1	Retinol dehydrogenase	F	5’	CAGCGTCAAAGGTCTAGGTG	3’
		R	5’	AGGGTTGATGGACTCGATTTC	3’
KUI73928.1	Retinol dehydrogenase	F	5’	CTGAAGCTAACCCACCTGATC	3’
		R	5’	GTACGAGCAGAGATCCAAGTG	3’

^a^F: Forward primer

^b^R: Reversed primer

## Additional files

Additional file 1: **Table S1.** The RNA quality for each sample. (XLSX 8 kb)

Additional file 2: **Table S2.** The count matrix for each sample. (XLSX 440 kb)

Additional file 3: **Figure S1.** KOG classification of *Vm*. The x-axis indicates the KOG function classification of the gene for *Vm*, and the y-axis indicates the number of genes. (PDF 6 kb)

Additional file 4: **Figure S2.** GO classification of *Vm*. The y-axis indicates the GO term at level 2 of genes for *Vm*, and the x-axis indicates the number of genes. (PDF 5 kb)

Additional file 5: **Figure S3.** KEGG classification of *Vm*. The y-axis indicates the metabolic pathways involved in the genes of *Vm*, and the x-axis indicates the number of genes. (PDF 5 kb)

## Abbreviations

CAT: Catalase
DEGs: Differentially expressed genes
GO: Gene Ontology
GST: Glutathione S-transferase
KEGG: Kyoto Encyclopedia of Genes and Genomes
PDA: Potato dextrose agar
PGs: Pectinase genes
ROS: Reactive oxygen species
SOD: Superoxide dismutase
*Sy* Hhs.015: *Saccharothrix yanglingensis* Hhs.015
*Vm*: *Valsa mali*
