# Supplementary figures and images for: Transcriptome analysis of Valsa mali reveals its response mechanism to the biocontrol actinomycete Saccharothrix yanglingensis Hhs.015
Source: BMC Microbiol. 2018 Aug 22;18:90. doi: 10.1186/s12866-018-1225-5 (PMC6106759; doi:10.1186/s12866-018-1225-5)

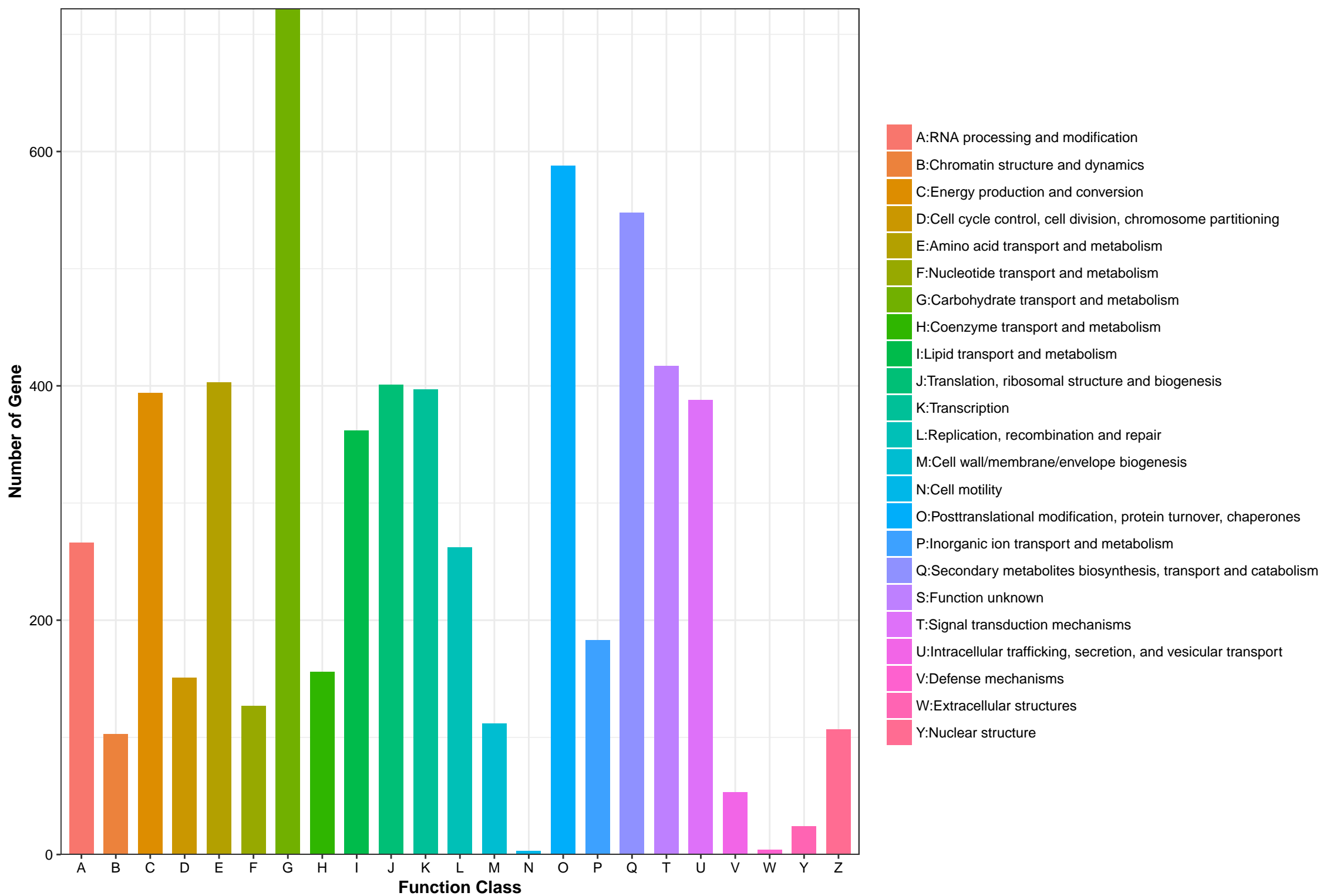

Supplement: Supplementary file 3 — Figure S1. KOG classification of Vm. The x-axis indicates the KOG function classification of the gene for Vm, and the y-axis indicates the number of genes. (PDF 6 kb) [file 12866_2018_1225_MOESM3_ESM.pdf]

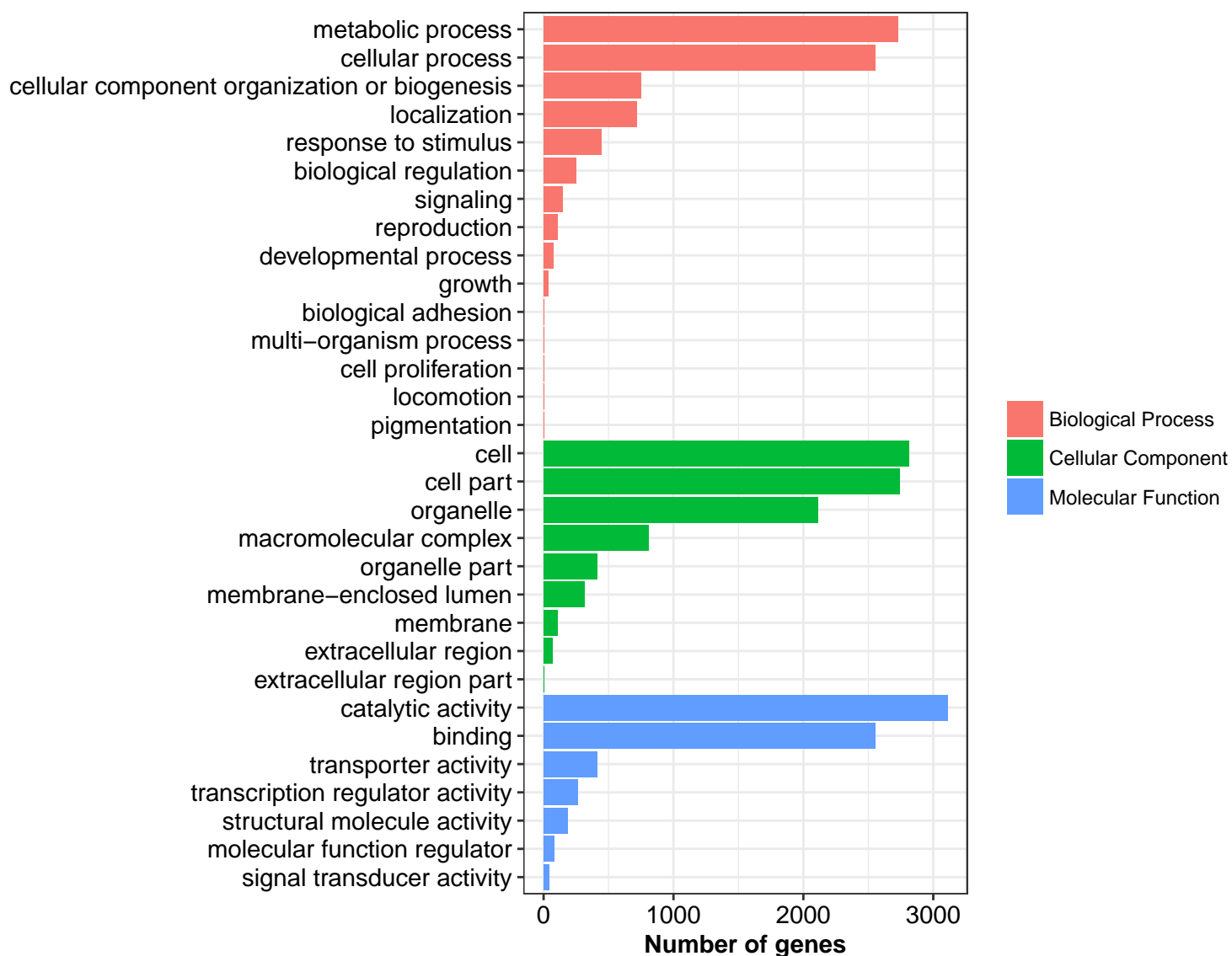

Supplement: Supplementary file 4 — Figure S2. GO classification of Vm. The y-axis indicates the GO term at level 2 of genes for Vm, and the x-axis indicates the number of genes. (PDF 5 kb) [file 12866_2018_1225_MOESM4_ESM.pdf]

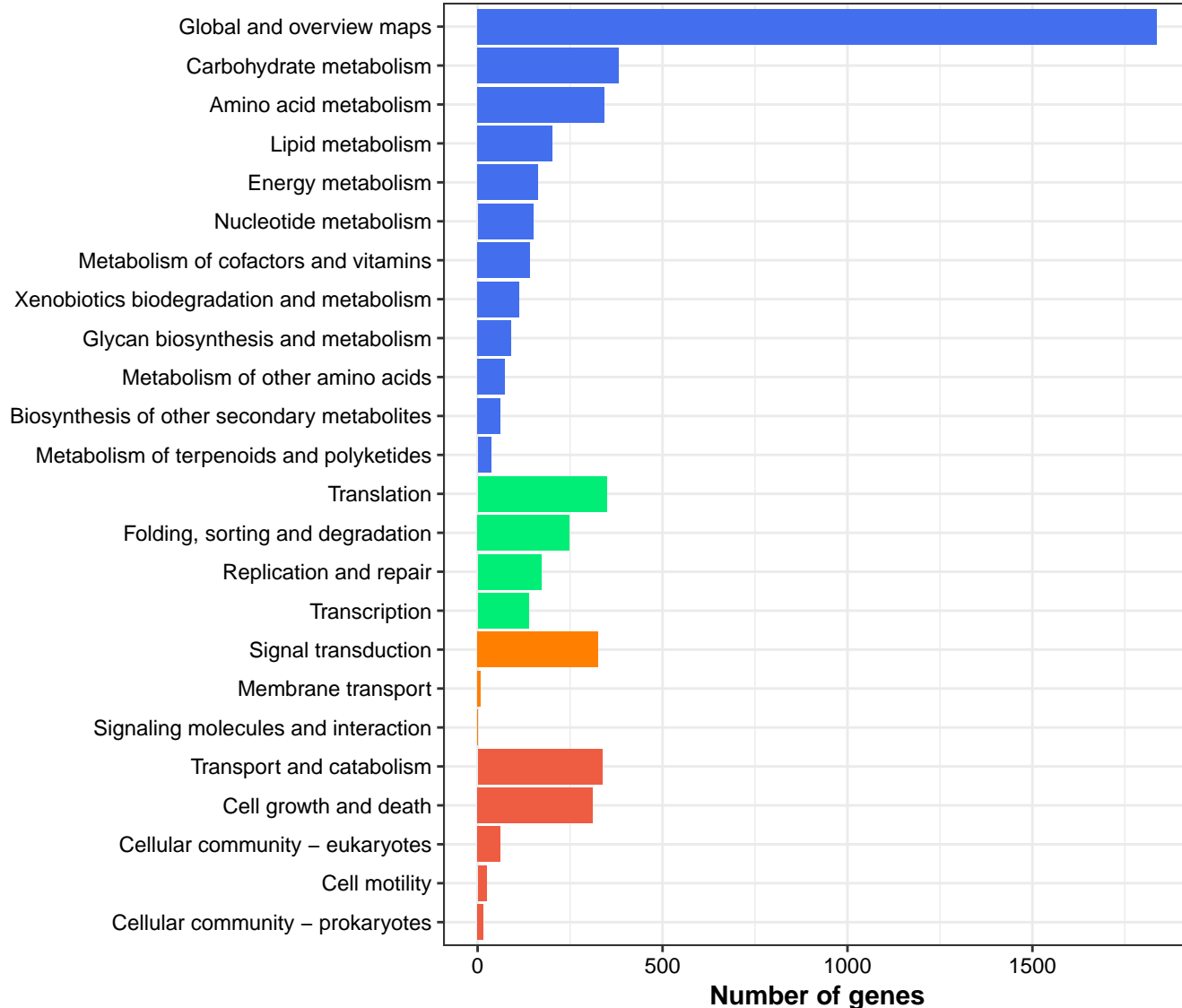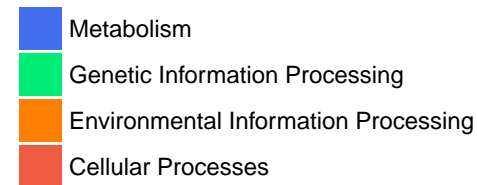

Supplement: Supplementary file 5 — Figure S3. KEGG classification of Vm. The y-axis indicates the metabolic pathways involved in the genes of Vm, and the x-axis indicates the number of genes. (PDF 5 kb) [file 12866_2018_1225_MOESM5_ESM.pdf]
